# mRNA vaccine design using the proteome of *Theileria annulata* through immunoinformatics approaches

**DOI:** 10.1128/msphere.00809-24

**Published:** 2025-05-01

**Authors:** Roohollah Fattahi, Behrooz Sadeghi Kalani

**Affiliations:** 1 Department of Laboratory and Clinical Sciences, Faculty of Veterinary Sciences, Ilam University, Ilam, Iran; 2 Department of Medical Microbiology, Faculty of Medicine, Ilam University of Medical Sciences, Ilam, Iran; University at Buffalo--Downtown Campus, Buffalo, New York, USA

**Keywords:** *Theileria annulata*, immunoinformatics, mRNA vaccine, epitopes, molecular docking

## Abstract

Theileriosis exerts a substantial impact on ruminants, resulting in significant economic losses within the animal husbandry sector. The current vaccine, a live attenuated parasite, has several limitations that hinder effective disease control. This study utilized immunoinformatics to prioritize potential vaccine candidates and pointed to the design of a novel mRNA vaccine against *Theileria annulata* using *in silico* methods. Nine antigenic proteins were selected using various software, and their epitopes were identified through immunoinformatics tools. These epitopes were assessed for their biological traits and homology. Their presentation by major histocompatibility complex (MHC) cells and other immune cells was analyzed using molecular docking techniques. A multi-epitope protein was then modeled and optimized, followed by structural and stability analyses of the mRNA vaccine construct. Finally, the immune response to the new vaccine was simulated. The identified epitopes were localized within the antigen-binding sites of their respective MHC alleles. The newly formulated vaccine demonstrated stability, exhibited no toxicity, and showed non-allergenic characteristics. It effectively elicited responses from both the humoral and cellular immune systems. The findings suggest that the desired engineered mRNA vaccine paves the way for the development of the deterrence of theileriosis. This potential merits additional exploration through rigorous laboratory experiments and subsequent clinical trials.

**IMPORTANCE:**

This study presents a cutting-edge approach in vaccine design against bovine theileriosis, a devastating disease affecting cattle globally. By leveraging immunoinformatics methodologies, a novel mRNA vaccine candidate was tailored using computational analyzes of *Theileria annulata* proteins. Antigenic protein identification, epitope evaluation, and structural optimization of the multi-epitope mRNA vaccine are pivotal advancements in vaccine development. Using computational modeling tools to predict immune responses enhances the efficiency and accuracy of vaccine design, potentially revolutionizing preventive strategies against bovine theileriosis. This research not only demonstrates the potential of immunoinformatics in vaccine innovation but also sheds light on a promising avenue for combating a significant livestock health concern, offering hope for more effective and targeted veterinary interventions.

## INTRODUCTION

Bovine theileriosis is a significant challenge for livestock in tropical and subtropical areas, affecting numerous crossbred and exotic cattle each year, and it is transmitted through ticks. Two significant species of the protozoan parasite genus *Theileria*, namely *T. parva* and *T. annulata*, are accountable for infections in cattle and pose a substantial threat to the global livestock industry (1, 2). *T. parva* is the causative agent of East Coast fever, a destructive disease endemic to sub-Saharan Africa that can result in mortality rates as high as 90% in infected cattle (3).

On the other hand, *T. annulata* is the main source of theileriosis in the world; this species causes significant economic losses through fever, anemia, and jaundice in affected animals (1, 4, 5).

Both *Theileria* species significantly impact the cattle industry by causing high mortality rates, reduced productivity, and necessitating costly control measures, resulting in estimated annual losses in the billions of dollars (6, 7). For instance, in India, the economic impact of blood parasite diseases in animals is predictable at approximately 498.7 million U.S. dollars (USD) annually, with bovine tropical theileriosis accounting for 384.3 million USD of this total. In Sub-Saharan Africa, over 1 million cattle deaths attributed to East Coast fever, caused by *T. parva*, occur each year, resulting in a loss of around 300 million USD annually. In endemic regions, the mortality rate for tropical theileriosis can reach 40%–90% (1, 6).

The management of tropical theileriosis primarily relies on the use of a live attenuated *T. annulata* schizont vaccine and the administration of hydroxynaphthoquinones, such as the drug uparvaquone (8, 9).

The use of live attenuated vaccines for the prevention of tropical theileriosis is widely adopted due to their ability to confer robust immunity. Nevertheless, this approach presents certain practical challenges (10–13). One potential drawback of live attenuated vaccines is the risk of presenting live vaccine organisms into regions where they are not endemic (1, 14). Another concern is that the live vaccine could perpetuate the life cycle of *T. annulata* in the field, potentially spreading the infection to unvaccinated animals through tick vectors. Additionally, live attenuated vaccines can result in the emergence of carrier animals within the cattle population (11, 15–17).

Recent studies have revealed that DNA vaccines targeting specific antigens can trigger whole immunity in cattle, protecting against *Theileria* infection. In contrast, subunit vaccines utilize purified *Theileria* proteins, such as TaSp, Tams1, SPAG1, and P67, or their peptides, to trigger an immune response (14, 18–21).

Regrettably, peptide-based subunit vaccines have a restricted capability to trigger an immune response, and there is a risk of DNA vaccines causing insertional mutagenesis in the host’s DNA. These issues pose challenges that need more research to overcome (8, 22–24).

Conversely, mRNA vaccines have proven to be more effective in addressing the security and usefulness concerns linked with DNA and peptide-based vaccines (25–28). Moreover, cell-free mRNA vaccines can be quickly and cost-effectively manufactured on a large scale. Another advantage is that a single mRNA vaccine can carry multiple antigens, improving the immune response against difficult pathogens. These vaccines have the capacity to induce in cooperation humoral and cellular immune responses, crucial for combating intracellular pathogens, like *Theileria* species. The adaptability, rapid development process, and ability to trigger a comprehensive immune response make mRNA vaccines as a promising strategy for preventing and managing theileriosis in cattle (24, 26–28).

Immunoinformatics is a bioinformatics specialty that connects computational analyzes with immunological data and tools. It plays a crucial role in designing vaccines by predicting the most suitable antigens, epitopes, carriers, and adjuvants essential for effective vaccines. Immunoinformatics has substantially decreased both the time and cost involved in developing vaccines (23, 27, 29, 30).

By utilizing these resources, scientists can accelerate the discovery and development of treatments and vaccines, enhancing their ability to combat infectious diseases more efficiently than ever before. Accordingly, This research seeks to create an innovative multivalent mRNA vaccine specifically designed to target *T. annulata*.

## MATERIALS AND METHODS

### Retrieval of protein sequences

The NCBI database was applied to retrieve amino acid sequences of *T. annulata*. The derived sequences were subsequently screened to identify proteins containing putative signal peptides using SignalP 5.0 (31). Furthermore, proteins with transmembrane domains were identified using transmembrane hidden Markov model (MHMM) (32). Subsequently, proteins predicted to be targeted to the mitochondria or apicoplast were excluded based on bipartite targeting predictions generated by TargetP (33).

### Prediction of immune cell epitopes

The ABCpred online tool (34) was exploited to forecast linear B-cell epitopes (LBL), employing an artificial neural network to offer insights into potential B-cell epitopes. The length of selected peptides for prediction was fixed at 16 amino acids, maintaining 80% specificity. An overlap filter was activated, and a threshold of 0.7 was applied to the submitted protein sequence. For predicting cytotoxic T lymphocyte (CTL) epitopes, the IEDB major histocompatibility complex class I (MHC I) web server was employed (35). The nine chosen proteins were introduced in FASTA format, utilizing the ANN 4.0 setting to predict 9-mer CTL epitopes. The complete bovine BoLA reference set was operated for this analysis.

### Bovine homology

The sequences of selected immune cell epitopes were analyzed using BLASTp against the cattle proteome (TaxID: 9913) in the NCBI database to prevent potential cross-reactions (36). Peptides with an E-value exceeding 0.05 were measured potentially non-homologous to cattle peptides.

### Assessment of epitope biological traits

Various online tools were utilized to assess the antigenicity, allergenicity, and toxicity of the chosen epitopes. The VaxiJen web server (37) predicts antigenicity by analyzing the physicochemical characteristics of epitopes without relying on sequence alignment, with a particular emphasis on proteins from parasites and using a threshold of 0.7. Similarly, the AllerTop V.2.0 web server (38), evaluates allergenicity using default settings. Additionally, the ToxinPred V.2.0 web server (39) assessed and quantified the toxicity of the epitopes by making all potential mutants using its default parameters. Only those epitopes that demonstrated high antigenicity while being non-toxic and non-allergenic were chosen for additional study.

### Molecular docking between cytotoxic T-cell lymphocyte (CTL) epitopes and MHC 1

The sequences of bovine MHC-I alleles, specifically BoLA-T2C (A8W3P5), BoLA-D18.4 (Q95396), BoLA-T2a (Q2V0B4), and BoLA-HD6 (O77972), were downloaded from the UniProt Database (40). As 3D crystal structures for these proteins were not accessible, valid models were constructed using homology modeling on the Swiss-Model server and refined by the protein structure refinement server (41, 42). The template 3D structure for all models was HLA-B*07:02 (P01889).

Molecular docking was conducted to investigate the binding affinity of selected CTL epitopes with the MHC I BoLA alleles. Three-dimensional structures of BoLA-T2C (A8W3P5), BoLA-D18.4 (Q95396), BoLA-T2a (Q2V0B4), and BoLA-HD6 (O77972) were obtained from the UniProt database and refined accordingly. Energy minimization of these structures was executed using Swiss-PDB Viewer (43). Selected epitopes were folded into their corresponding three-dimensional conformations using the PEP-FOLD 4 server (44). These models underwent additional energy minimization using Swiss-PDB Viewer prior to docking. Docking procedures were executed using the ClusPro 2.0 server and LigPlot^+^ used to visualize interactions between the epitopes and MHC alleles (45).

### Design of the vaccine construct

The epitopes characterized by high antigenicity, the absence of allergenicity and toxicity, and non-similarity to the cattle proteome were nominated as the most favorable candidates for vaccine development. An mRNA vaccine construct has been planned following a specific sequence order from the N-terminus to the C-terminus. This sequence includes a adapted cap structure (m7GCap), a 5′ untranslated region (5′ UTR), and a Kozak sequence to augment translation efficiency. The coding region initiates with a signal peptide (tPA), which is linked to an adjuvant component via an EAAAK linker. Most subunit vaccines typically incorporate an adjuvant to effectively activate innate immunity and make a vigorous immune response. In this study, RpfE was identified as a suitable adjuvant for the vaccine (25, 26, 28, 46).

### Forecasting the antigenic potential, allergenic potential, toxicity, and physicochemical characteristics of the vaccine formulation

The antigenicity and allergenicity of the mRNA vaccine construct were predicted using VaxiJen v2.0 and AllerTOP, respectively. VaxiJen 2.0 relies on the physicochemical characteristics of the vaccine for its predictions, whereas ANTIGENpro employs machine learning algorithms along with microarray analysis data. The amino acid sequence of the developed mRNA vaccine, not including the tPA and MITD sequences, was input into these tools. AllerTOP 2.0 was used to assess allergenicity, and the ToxinPred server was employed to forecast toxicity (37–39). Furthermore, the ProtParam online web server was utilized to predict a range of physicochemical characteristics of the desired vaccine (37).

### *In silico* immune simulation

The immunological responses elicited by the mRNA vaccine were predicted through *in silico* immune simulations utilizing the C-ImmSim server (47, 48). This server initiates an immune response by integrating epitopes with lymphocyte receptors. In accordance with established vaccine dosing protocols, three doses of 1,000 vaccine units were directed over a 4-week period. All parameters were maintained at their default settings, with injections occurring at time points 1, 84, and 168 h.

### Optimization of codons for the desired vaccine

To improve mRNA expression in the bovine cell vaccine, the GenSmart tool from GenScript was employed for codon optimization. The modified sequence was then assessed for quality using rare codon analysis and evaluated for translation efficiency through the codon adaptation index (CAI). Identifying rare tandem codons further informed the optimization process, enhancing the vaccine’s overall expression and efficacy (49).

### Prediction of secondary structure for the designed mRNA vaccine

To evaluate the secondary structure of the mRNA vaccine, we used the RNAfold tool from the Vienna RNA Package 2.0 (50).

### Prediction and validation of secondary and tertiary structures for the vaccine

For predicting the secondary structure of the studied vaccine, the PSIPRED server was employed. This server is well-regarded for its precision in forecasting protein secondary structures, utilizing two feed-forward neural networks to process data generated from PSI-BLAST (51). To determine the 3D structure of the peptide sequence, we turned to the Robetta server, which produced five potential structural models (52, 53). The finest model investigated by ProSA-web, PROCHECK, and ERRAT servers (26, 54–56).

### Prediction of the conformational B-cell epitopes

To this, the ElliPro online server was conducted. This tool leverages the geometric characteristics of the three-dimensional model to predict these epitopes and has been shown to surpass other existing tools in accuracy, achieving the highest area under the curve (AUC) score of 0.732 across a range of protein models (57).

### Investigation of molecular interaction of the vaccine and Toll-like receptors (TLRs)

To evaluate potential interactions between the target mRNA vaccine and TLR 2, 3, 4, and 9, we employed the ClusPro server (45). The sequences for bovine TLR-2 (Q95LA9), TLR-3 (Q5TJ59), and TLR-4 (Q9GL65) were sourced from the UniProt Database (40). Given the lack of available crystal structures for these proteins, we generated a reliable model via the Swiss-Model server, which was additional refined using the Protein Structure Refinement Server. We conducted docking analysis on the three-dimensional structures of both the vaccine and the bovine TLRs (TLR-2: Q95LA9, TLR-3: Q5TJ59, TLR-4: Q9GL65, TLR-9: Q5I2M5) utilizing the PIPER docking algorithm.

### Molecular dynamics simulation

To authorize the physical dynamics of atoms and molecules within the complexes formed by bovine TLR-2, TLR-3, TLR-4, and TLR-9 in conjunction with the vaccine, molecular dynamics simulations were carried out using the iMODS server. We selected complexes with the most favorable binding energies for this analysis to ensure precision. The stability and movement of atoms and molecules within these complex structures were assessed through the iMODS server (58).

## RESULTS

### Retrieval of protein sequences

From an extensive data set comprising 8,887 protein sequences of *T. annulata* obtained from the NCBI database, we identified 408 proteins that possess putative signal peptides indicative of potential secretion. Among these, 31 proteins were further characterized as containing transmembrane domains, suggesting their involvement in membrane-associated functions. To refine our analysis, we employed the TargetP algorithm to filter out proteins localized to mitochondria or apicoplasts, which are not relevant to our study focus. This rigorous filtration process ultimately led to the identification of nine specific protein sequences of *T. annulata* that warrant further investigation. The accession numbers for these proteins in the NCBI database are as follows: XP954696.1, XP955218.1, XP955339.1, XP955208.1, XP954872.1, XP954977.1, XP954655.1, XP955123.1, and XP_954937.1.

### Prediction and estimation of B-cell epitopes

In our analysis of B-cell epitopes, we identified a total of 16 epitopes that exhibited high antigenicity, with scores exceeding 0.7 as determined by VaxiJen. These epitopes were carefully selected based on their favorable properties, including non-allergenic and non-toxic characteristics, making them promising candidates for inclusion in the vaccine construct (refer to Table 1 for detailed information).

**TABLE 1:** Cell type and sequence of the epitopes in this study

Protein name	Function	CTL epitopes	MHC I binding alleles	LBL epitopes
XP_954977.1	Sporozoite surface antigen, putative	KLLGSGFEV	BoLA-T2C	SQTGLGPSGSHAQQDP DGTTTGPGGNGEGGKD
		TTTTTSSGK	BoLA-T2a	
		KPAELGPSL	BoLA-T2C	
XP_954872.1	Uncharacterized protein TA05495	TLWAYKVWL	BoLA-T2C	TERIEDPRTEINTATH
		WLAVVVVLL	BoLA-T2C	
		SSFLEHSSK	BoLA-T2a	
XP_955208.1	(Zn) transporter protein	RGFIIFAPI	BoLA-HD6	GFGIIAHKWAESILLT LVLIDFLPGENYHETK
		AISGALLSL	BoLA-T2C	
		SEILKTAYI	BoLA-HD6	
		TSLLSDSFK	BoLA-T2a	
XP_955339.1	*Theileria*-specific hypothetical protein	TLKQTGRVL	BoLA-T2C	YGNITLKQTGRVLDFK GRVLDFKVRSTFEANK
		ISLMLFLIK	BoLA-T2a	
		KGRYGNITL	BoLA-HD6	
XP_955218.1	Uncharacterized protein TA05025	KLQGVGENL	BoLA-T2C	HSLIISSKPSKNYNSK TERIEDPRTEINTATH
		WLLVLVVEL	BoLA-T2C	
		TVFLHKTFK	BoLA-T2a	
XP_954696.1	*Theileria*-specific sub-telomeric protein	IEYRNKGVL	BoLA-HD6	LGPGILGPAPGPLREP
		TQPYPPAPY	BoLA-D18.4	
		DIQWNDIFL	BoLA-T2C	
XP_954937.1	Uncharacterized protein TA03530	NFTHFLYTL	BoLA-T2C	TERIEDPRTEINTATH SRSIYSSVNPRIERDS
		IQIFFRQSL	BoLA-HD6	
		LLIPFSLYL	BoLA-T2C	
XP_955123.1	Uncharacterized protein TA04545	MLLYNKFLI	BoLA-HD6	TERIEDPRTEINTATH KEPTFFSLLFHPKVKE
		FLILINLNI	BoLA-HD6	
		LQANPNVEK	BoLA-D18.4	
XP_954655.1	Kelch-domain containing protein	WTRLKSGQF	BoLA-HD6	LYVIGGHNDNGTLNTI YKTYKTYAEFTLLSDE
		LLLAHCVKI	BoLA-HD6	

### Prediction and estimation of the CTL epitopes

For the prediction of CTL epitopes, we identified 28 epitopes that met stringent criteria for selection. These criteria included high antigenicity, non-allergenicity, non-toxicity, and a lack of significant homology with human proteins, thereby reducing the risk of adverse immune responses. The selected CTL epitopes are also included in Table 1 for reference.

### Molecular docking of CTL epitopes with bovine MHC alleles

**Fig 1:**
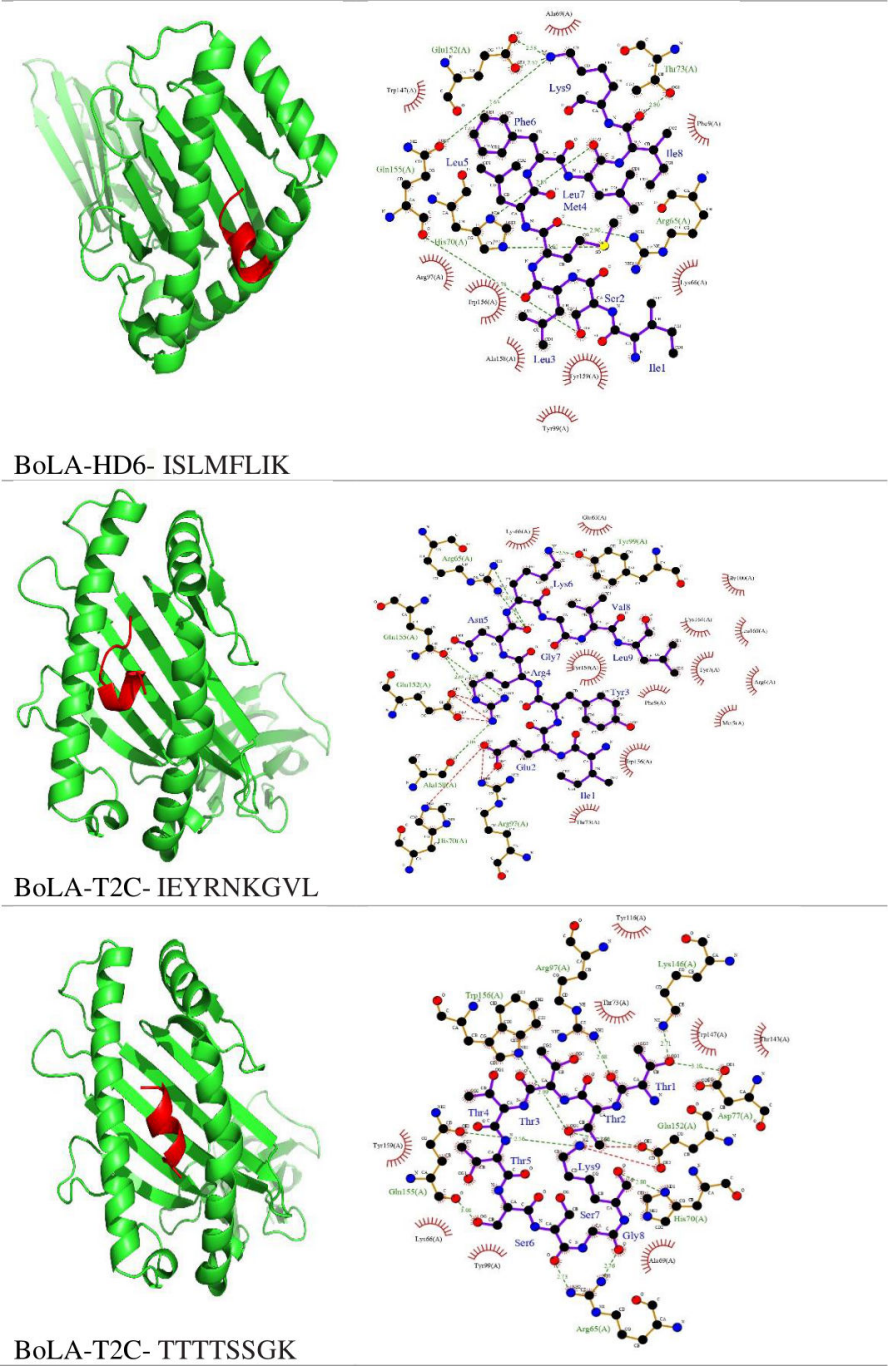
Results of molecular docking of epitopes and their corresponding MHC alleles. The left side of the figure illustrates a cartoon representation of the docking results, while the right side displays the interactions between the epitopes and MHC alleles as revealed by LigPlot+.

All 28 cytotoxic T lymphocyte epitopes were modeled using the PEP-FOLD 4 server and subsequently docked with their corresponding bovine MHC-I alleles. Among these, the CTL epitopes FLILINLNI, TLWAYKVWL, WLAVVVVLL, LLIPFSLYL, and LLLAHCVKI demonstrated the highest binding affinities to the bovine MHC-I allele, with energy values recorded at −1,047.4, –992.4, −958.0, –936.2, and −912.8 kcal/mol, respectively (refer to Table 2 for detailed binding affinity data). The binding interactions are visually represented in Fig. 1, showcasing the robust docking models of CTL epitopes interacting with bovine MHC-I alleles. To further elucidate the nature of these interactions, we employed the LigPlot^+^ tool for analysis, with the results depicted in Fig. 1.

**TABLE 2:** Molecular interface analysis of some CTL epitopes with their conforming MHC alleles

Type of T lymphocyte	Epitope	MHC alleles	Uniprot ID of MHC allele	Binding affinity (kcal/mol)
**CTL**	FLILINLNI	BoLA-HD6	O77972	−1,047.4
	TLWAYKVWL	BoLA-T2C	A8W3P5	−992.4
	WLAVVVVLL	BoLA-T2C	A8W3P5	−958
	LLIPFSLYL	BoLA-T2C	A8W3P5	−936.2
	LLLAHCVKI	BoLA-HD6	O77972	−912.8

**Fig 2:**
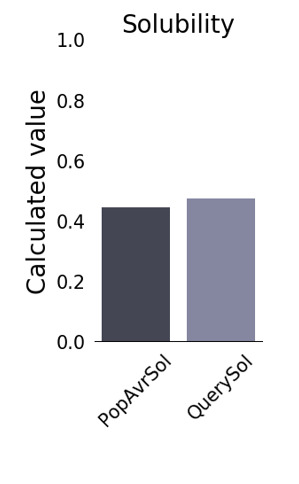
Solubility index of the designed vaccine.

### Vaccine construct design

In our study, we meticulously selected a total of 28 cytotoxic T lymphocyte (CTL) epitopes and 16 B-cell epitopes based on comprehensive immunogenic assays. The selected epitopes were strategically linked using specific peptide linkers to ensure optimal structural integrity and immunogenic potential. The CTL epitopes were interconnected using AAY linkers, while a GGTGG linker was employed to connect the terminal end of the CTL epitopes to the initial B-cell epitopes. For the linkage of the B-cell epitopes, a KK linker was utilized. The final structure of our mRNA vaccine was carefully designed and comprises the following components, arranged sequentially from the N-terminal to the C-terminal (the linkers have been shown in bold): 5′ m7GCap, 5′UTR, Kozak sequence, signal peptide (tPA), **EAAAK**, adjuvant (RpfE)–AAY–KLLGSGFEV-AAY-AQQDPGVGV-AAY-TTTTTSSGK-AAY-KPAELGPSL-AAY-TLWAYKVWL-AAY-WLAVVVVLL-AAY-SSFLEHSSK-AAY-RGFIIFAPI-AAY-AISGALLSL-AAY-SEILKTAYI-AAY-TSLLSDSFK-AAY-TLKQTGRVL-AAY -ISLMLFLIK-AAY-KGRYGNITL-AAY-KLQGVGENL-AAY-WLLVLVVEL-AAY-TVFLHKTFK-AAY-IEYRNKGVL-AAY-TQPYPPAPY-AAY-DIQWNDIFL-AAY-NFTHFLYTL-AAY-IQIFFRQSL-AAY-LLIPFSLYL-AAY-MLLYNKFLI-AAY-FLILINLNI-AAY-LQANPNVEK-AAY-WTRLKSGQF-AAY-LLLAHCVKI-GGTGG-SQTGLGPSGSHAQQDP-KK-DGTTTGPGGNGEGGKD-KK-TERIEDPRTEINTATH-KK-GFGIIAHKWAESILLT-KK-LVLIDFLPGENYHETK-KK-YGNITLKQTGRVLDFK-KK-GRVLDFKVRSTFEANK-KK-HSLIISSKPSKNYNSK-KK-TERIEDPRTEINTATH-KK-LGPGILGPAPGPLREP-KK-TERIEDPRTEINTATH-KK-SRSIYSSVNPRIERDS-KK-TERIEDPRTEINTATH-KK-KEPTFFSLLFHPKVKE-KK-LYVIGGHNDNGTLNTI-KK-YKTYKTYAEFTLLSDE-KK-MITD sequence–stop codon–3′ UTR–poly(A) tail.

This comprehensive design ensures a robust immune response by integrating multiple epitopes that target both CTL and B-cell pathways, thereby enhancing the potential efficacy of the mRNA vaccine against the targeted pathogen.

### Assessment of biological and physicochemical properties of the vaccine construct

The analysis of the vaccine construct revealed several promising biological and physicochemical properties. Notably, the construct achieved a significant antigenicity score of 1.0045 as determined by VaxiJen, indicating its potential effectiveness as an immunogen. Importantly, the vaccine was classified as non-allergenic and non-toxic, which is essential for ensuring safety in potential clinical applications. Physicochemical characterization demonstrated that the molecular weight of the vaccine construct was calculated to be 75,693.85 Da (refer to Table 3).

**TABLE 3:** The physicochemical possessions of the proposed mRNA vaccine

Property	Measurement	Indication
Total number of amino acids	683	Appropriate
Molecular weight	75,693.85 Da	Appropriate
Formula	C_3479_H_5413_N_897_O_987_S_3_	–^*a*^
Theoretical pI	9.46	base
Total number of negatively charged residues (Asp + Glu)	65	–
Total number of positively charged residues (Arg + Lys)	93	–
Total number of atoms	10779	–
Instability index (II)	33.31	Stable
Aliphatic index (A.I.)	86.41	Thermostable
Grand average of hydropathicity (GRAVY)	−0.300	Hydrophilic
Antigenicity (using VaxiJen)	1.0045	Antigenic
Antigenicity (using ANTIGENpro)	0.614174	Antigenic
Allergenicity (using AllerTop 2.0)	Non-allergenic	Non-allergenic
Toxicity (ToxinPred)	Non-toxic	Non-toxic

^*a*^ "–," no specific significance.

The estimated half-life of the construct varied depending on the biological system: approximately 4.4 h in mammalian reticulocytes *in vitro*, over 20 h in yeast *in vivo*, and exceeding 10 h in *Escherichia coli in vivo*. These findings suggest that the vaccine is likely to maintain stability and efficacy over time.

The instability index was calculated at 33.31, indicating favorable stability characteristics for the vaccine construct. Additionally, an aliphatic index of 86.41 categorizes the protein as thermostable, suggesting resilience under varying temperature conditions. The theoretical isoelectric point (pI) was determined to be 9.46, which may influence the vaccine’s solubility and interaction with immune components.

The grand average of hydropathicity (GRAVY) index was calculated at −0.300, reflecting a polar nature with a strong affinity for water, which implies high solubility—an advantageous property for vaccine formulations. Analysis of the amino acid composition revealed a total of 65 negatively charged residues (Asp + Glu) and 93 positively charged residues (Arg + Lys), contributing to the overall charge balance of the protein.

The chemical formula of the vaccine was determined to be C_3479_H_5413_N_897_O_987_S_3_, providing insight into its molecular composition. According to Fig. 2, the solubility of the vaccine construct was quantified using QuerySol (scaled solubility value), yielding a measurement of 0.477. Collectively, these results indicate that the multi-epitope mRNA vaccine developed in this study is a promising candidate for combating *T. annulata*.

### *In silico* immune response simulation against the vaccine

**Fig 3:**
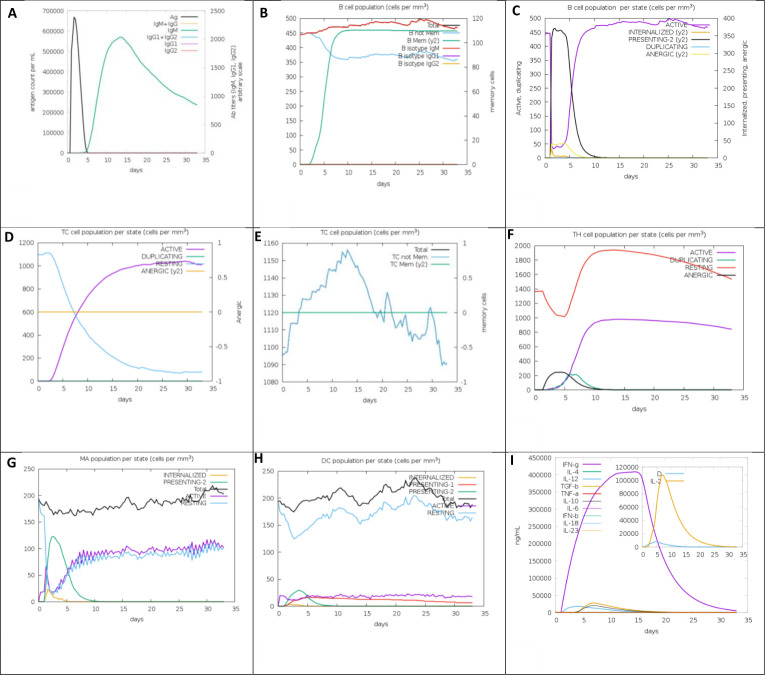
*In silico* immune imitation against the mRNA vaccine retrieved from the C-ImmSim server. (**A**) The antibody production. (**B**) The B-cell population. (**C**) The B-cell population. (**D**) T-cell population. (**E**) T-cell population. (**F**) The helper T-cell population. (**G**) Macrophage population. (**H**) Dendritic cell population. (**I**) Cytokine and interleukin creation with Simpson index of the immune response.

The *in silico* simulation results demonstrated that the constructed vaccine effectively activated the innate immune system and elicited robust adaptive immune responses. Specifically, the data indicated that the vaccine construct successfully stimulated B cells to produce elevated levels of IgM (see Fig. 3A), which is indicative of potential immune memory formation. Additionally, significant responses were observed in both T-helper cells and cytotoxic T lymphocytes (CTLs), as illustrated in Fig. 3B and D. The population of active cytotoxic T lymphocytes exhibited a gradual increase by day 10 post-vaccination and remained elevated thereafter, suggesting sustained immune activation. This increase in both CTL and helper T-cell populations was accompanied by notable memory formation. Moreover, macrophage activity was enhanced, contributing to overall immune efficacy, while dendritic cell activity remained consistently high throughout the simulation period. The proposed vaccine also induced elevated levels of key cytokines, including IFN-γ and IL-2, which are critical for orchestrating immune responses. Epithelial cells, integral components of innate immunity, showed similar enhancements. Finally, the Simpson index (D) exhibited a low value, indicating a diverse immune response profile. In summary, these findings suggest that the multi-epitope mRNA vaccine construct generates a robust and multifaceted immune response against *T.annulata*, highlighting its potential as an effective immunotherapeutic strategy.

### Codon improving of the mRNA construct

The results indicated a codon adaptation index (CAI) of 0.71 for our vaccine with a coding sequence (CDS) length of 2,049 nucleotides. Moreover, the optimized construct exhibited an average GC content of 50.37%. These findings, illustrated in Fig. 5, collectively indicate that this optimized mRNA vaccine construct is well-suited for efficient expression within cattle cells.

### Prediction of the secondary structure of the mRNA vaccine

**Fig 4:**
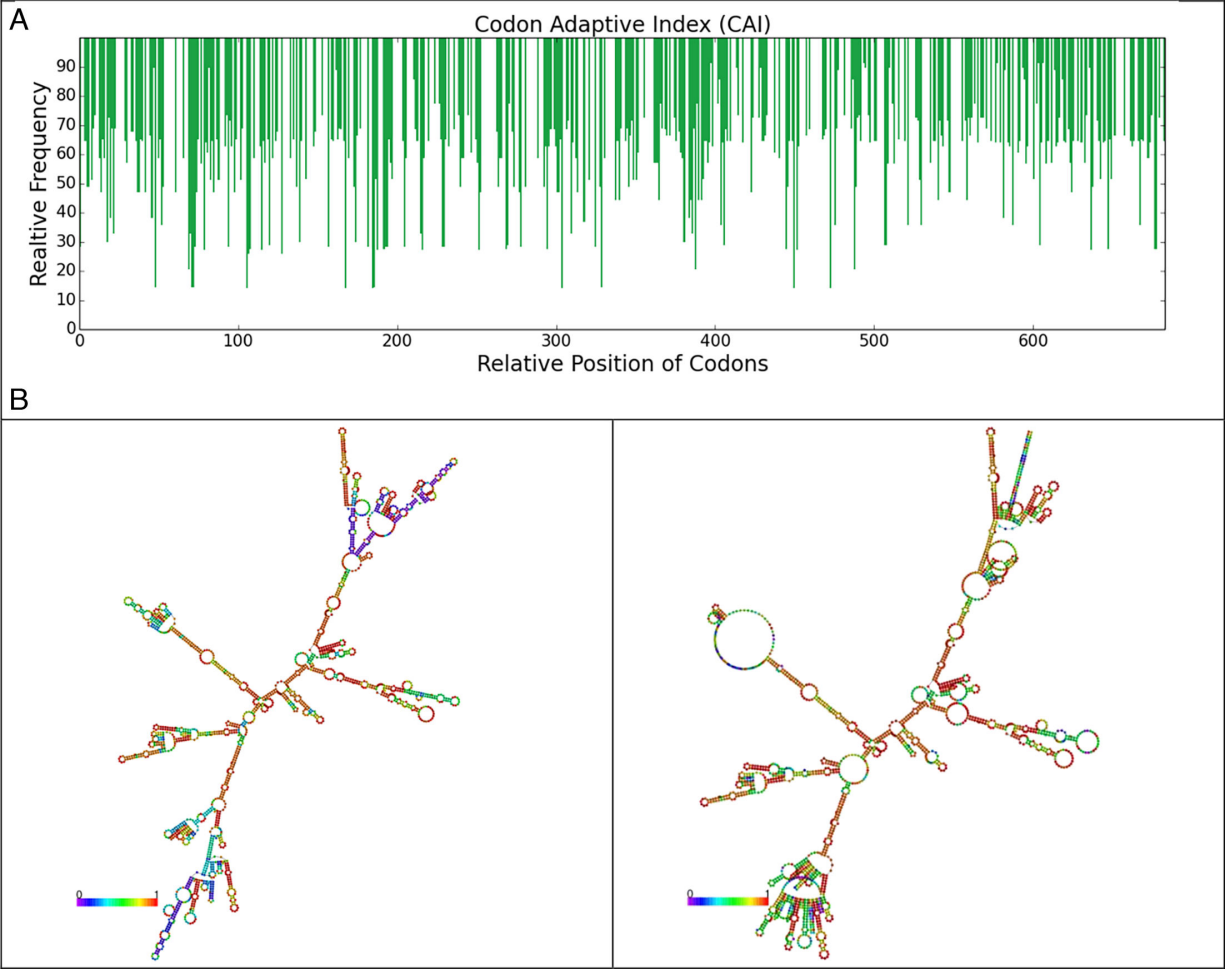
Codon optimization (A) and mRNA vaccine structure prediction (B).

The secondary structure of the mRNA sequence, yielding a minimum free energy (MFE) score of −669.79 kcal/mol. This analysis suggests that the mRNA vaccine construct is expected to maintain stability throughout the manufacturing process. Furthermore, the secondary centroid structure showed a free energy of −539.77 kcal/mol. These findings indicate that the mRNA vaccine construct possesses a stable configuration, allowing for efficient production. The predicted secondary structure of the multiepitope vaccine mRNA is presented in Fig. 4.

### Valuation of secondary and tertiary structures of the translated construct

**Fig 5:**
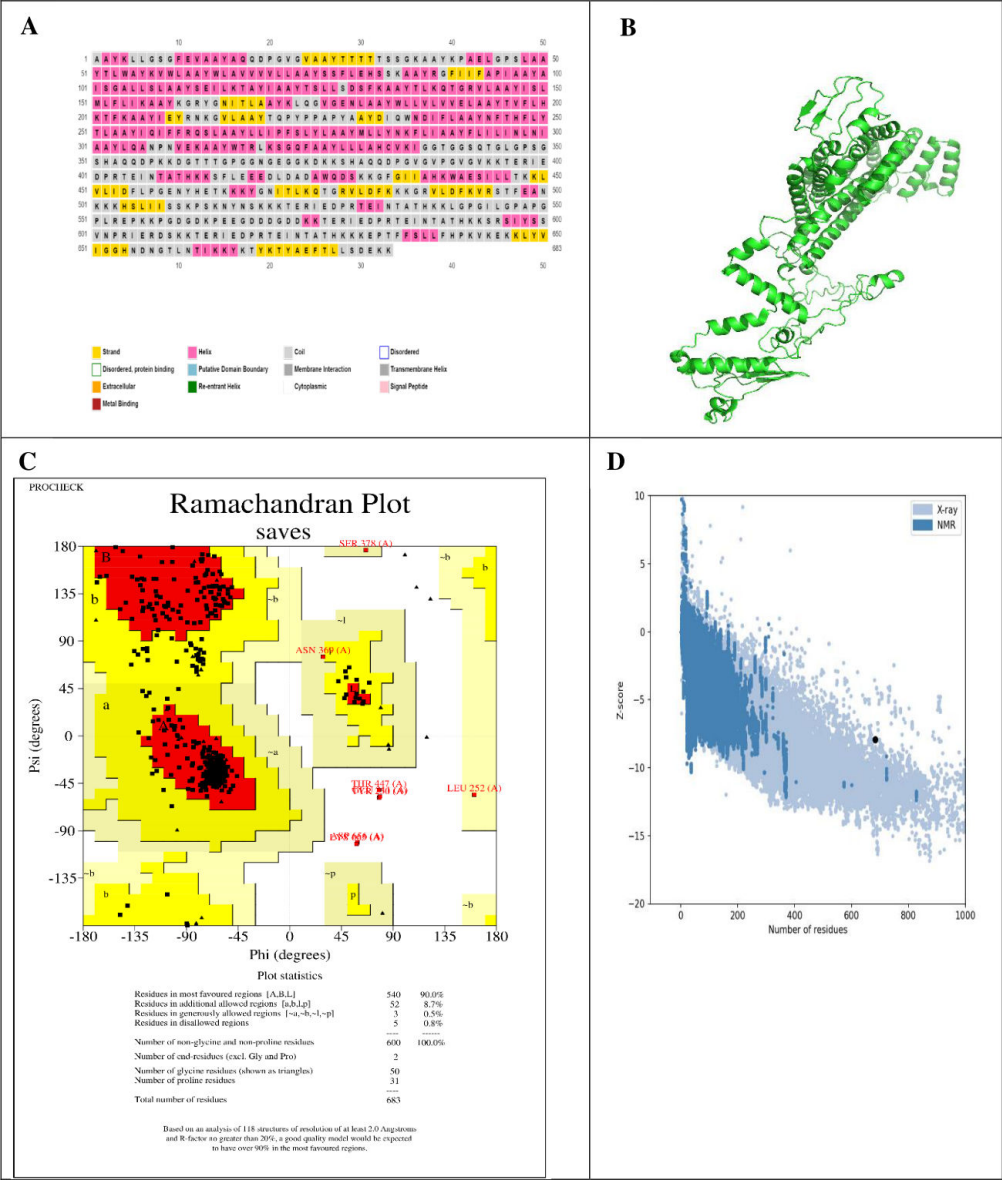
Structure prediction and validation of the peptide vaccine construct. (**A**) Secondary structure; (**B**) tertiary structure of the peptide using the Robetta server; (**C**) Ramachandran plot analysis using the PROCHECK server; (**D**) Z-score analysis using ProSA webserver.

**Fig 6:**
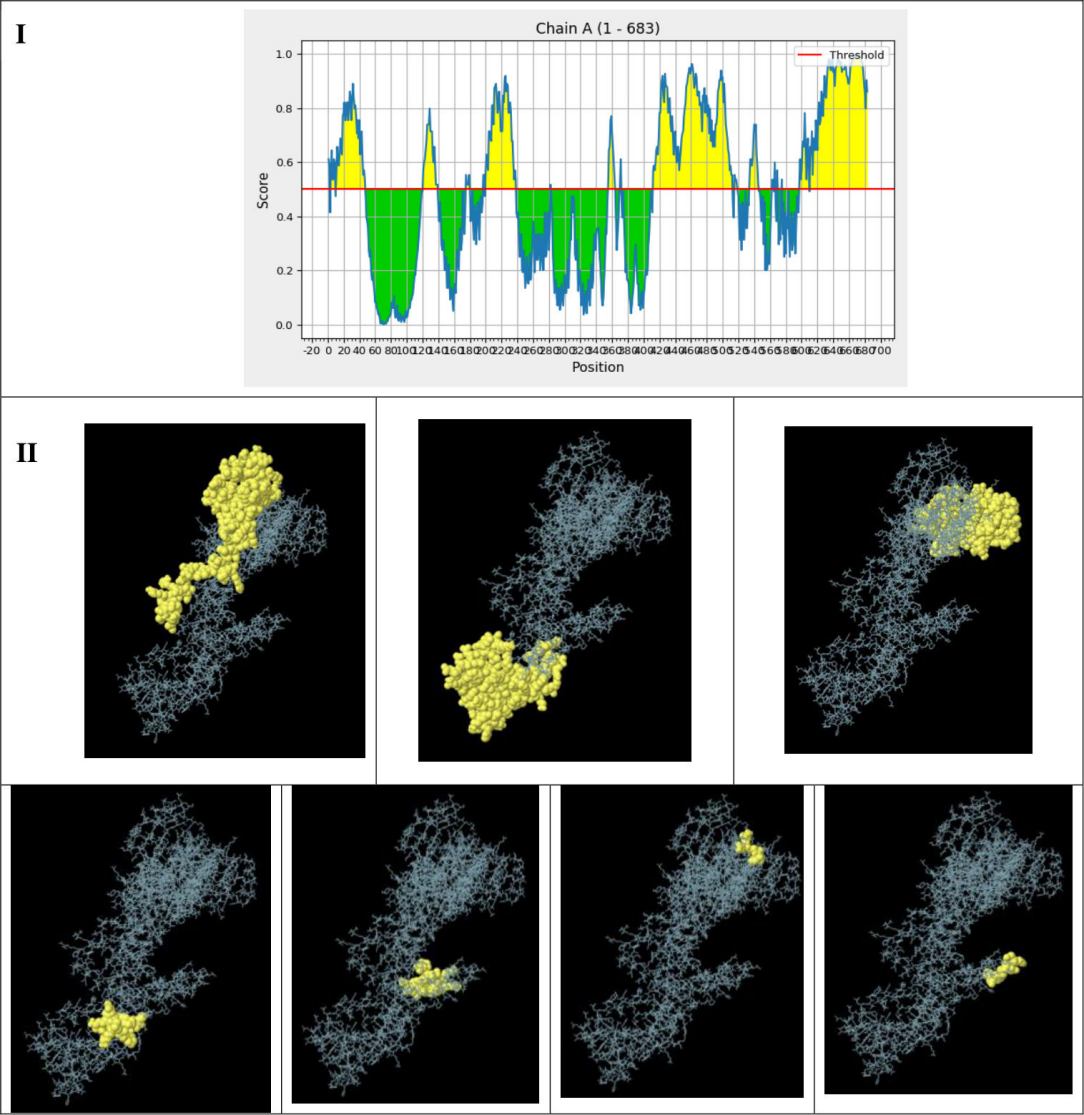
Presents the seven anticipated conformational B-cell epitopes identified using the ElliPro tool within the IEDB database. (**I**) Displays a two-dimensional representation illustrating the locations of these conformational B-cell epitopes. (II) Shows three-dimensional models of the B-cell epitopes, where the spheres indicate the conformational B-cell epitopes. The details regarding the residues and their corresponding scores are as follows (from top left to bottom right): 87 residues with a score of 0.846, 109 residues with a score of 0.754, 104 residues with a score of 0.705, 15 residues with a score of 0.597, 18 residues with a score of 0.55, 5 residues with a score of 0.531, and 5 residues with a score of 0.505.

RNA structure analysis revealed a structural composition comprising 17% alpha-helix, 32% beta strand, and 33% coil, as illustrated in Fig. 6. The predominant alpha helices in the structure are depicted in Fig. 5A. The Rosetta server facilitated the prediction of the three-dimensional structure of the vaccine construct, as shown in Fig. 5B. This process resulted in five distinct 3D models, each ranked according to their C-score. Following this, the tertiary structure of the vaccine underwent refinement using the Galaxy Refine online server and was subsequently corroborated with a Ramachandran plot (Fig. 5C). The Ramachandran plot demonstrated that 90% of the residues resided within the most favored region, indicating a robust model, with 8.7% in the additionally permitted region, 0.5% in the generously allowed region, and merely 0.8% in the disallowed region. The overall quality factor was computed to be 83.8124.

Moreover, a ProSA-web analysis yielded a negative Z-score of −7.94, suggesting a high level of consistency within the 3D protein model (Fig. 5D). In summary, these evaluations indicate that the mRNA vaccine construct exhibits stability, accuracy, and consistency, potentially increasing its effectiveness as a vaccine.

### Conformational B-cell epitope forecast

The analysis revealed seven non-contiguous B-cell epitopes, with prediction scores ranging from 0.505 to 0.846 across a total of 346 residues. The two-dimensional (2D) and three-dimensional (3D) representations of these conformational B-cell epitopes are illustrated in Fig. 6I and II, respectively. These epitopes are critical for inducing humoral immunity and promoting the production of antibodies against foreign antigens.

### Interaction with TLR and vaccine design

**Fig 7:**
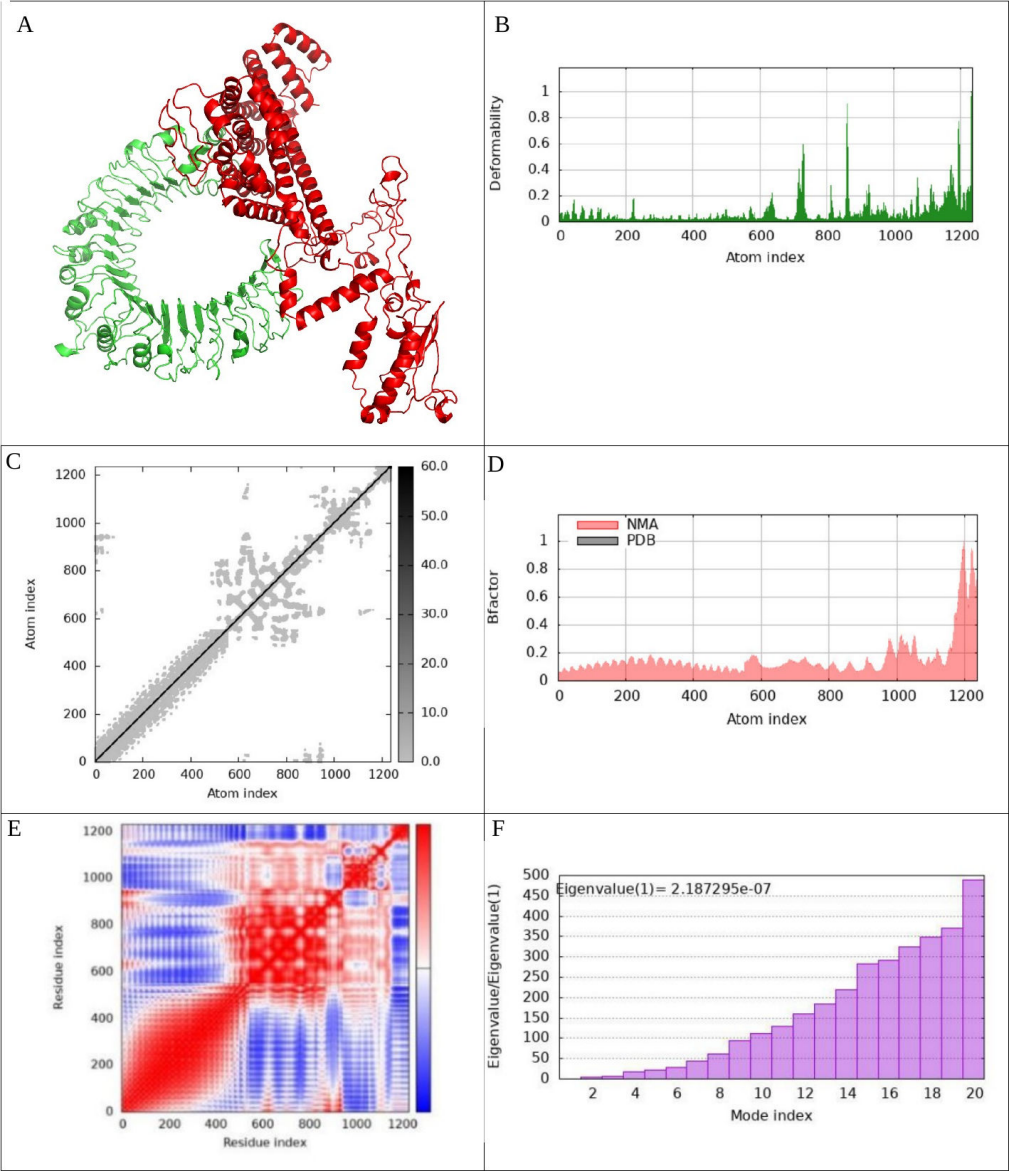
This figure encompasses various analytical components related to the vaccine–TLR2 docked complex generated via the Cluspro server. It includes (**A**) the docked complex; (**B**) a deformability graph; (**C**) an elastic network model constructed using the iMODS server; (**D**) a B-factor graph; (**E**) a covariance matrix; (**F**) eigenvalues of the vaccine-TLR2 complex; (**G**) receptor–ligand interactions analyzed with the PDBsum webserver; and (**H**) receptor–ligand interactions visualized using the LigPlot^+^. This comprehensive overview highlights the molecular dynamics simulation, normal mode analysis, and receptor–ligand interactions pertaining to the vaccine–TLR2 complex.

**Fig 8:**
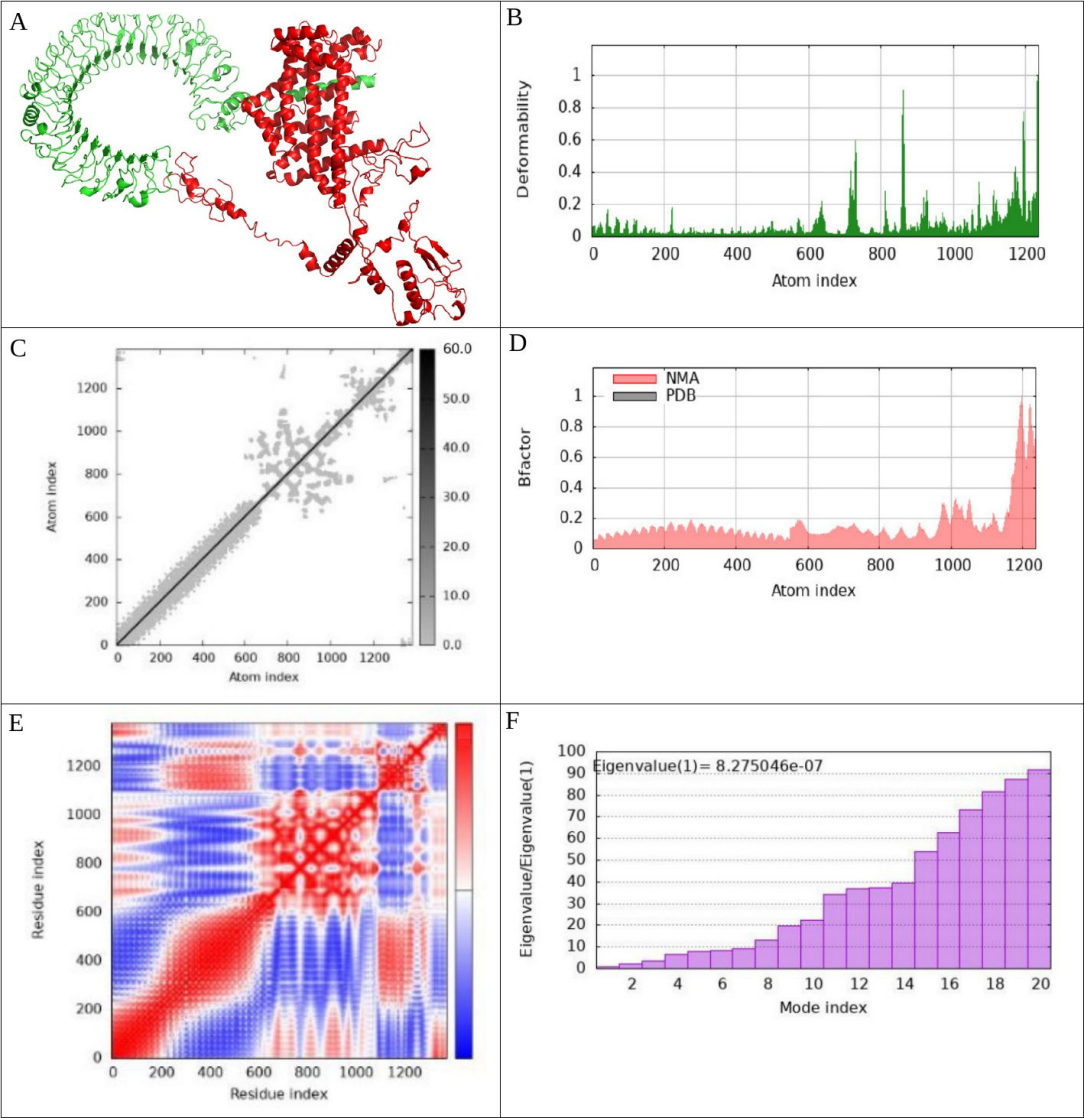
Examination of molecular dynamics simulations, normal mode analysis, and receptor–ligand interactions: This figure presents the following components related to the vaccine–TLR3 complex: (**A**) the docked structure generated by the Cluspro server; (**B**) a graph illustrating the deformability of the complex; (**C**) an elastic network model created using the iMODS server; (**D**) a representation of the B-factor graph; (**E**) a covariance matrix; (**F**) the eigenvalue corresponding to the vaccine–TLR3 complex; (**G**) analysis of receptor–ligand interactions conducted using the PDBsum webserver; and (**H**) visualization of these interactions through the LigPlot^+^. Collectively, this figure provides an in-depth analysis of molecular dynamics simulations, normal mode analysis, and receptor–ligand interactions pertaining to the vaccine–TLR3 complex, encompassing various analytical dimensions.

**Fig 9:**
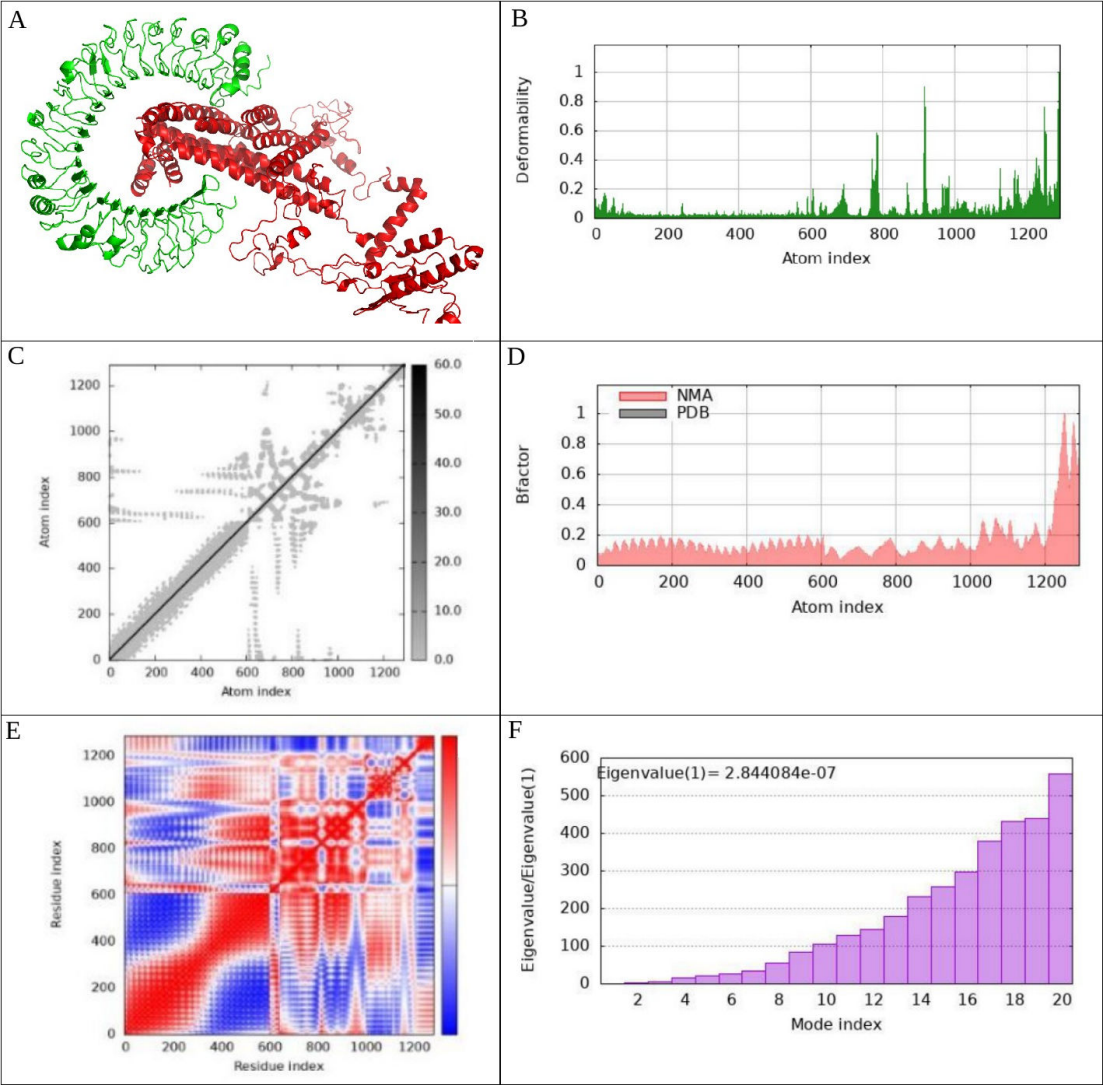
Examination of molecular dynamics simulations, normal mode analysis, and receptor-ligand interactions. This figure includes a detailed analysis of the vaccine–TLR4 complex, featuring the following: (**A**) the docked configuration produced via the Cluspro server; (**B**) a graph that depicts the deformability of the complex; (**C**) an elastic network model developed with the iMODS server; (**D**) a representation of the B-factor graph; (**E**) a covariance matrix; (**F**) the eigenvalue linked to the vaccine–TLR4 complex; (**G**) examination of receptor–ligand interactions performed using the PDBsum webserver; and (**H**) visualization of receptor–ligand interactions using the LigPlot^+^ webserver. This figure offers a thorough overview of molecular dynamics simulations, normal mode analysis, and receptor–ligand interactions for the vaccine–TLR4 complex, emphasizing various analytical elements.

**Fig 10:**
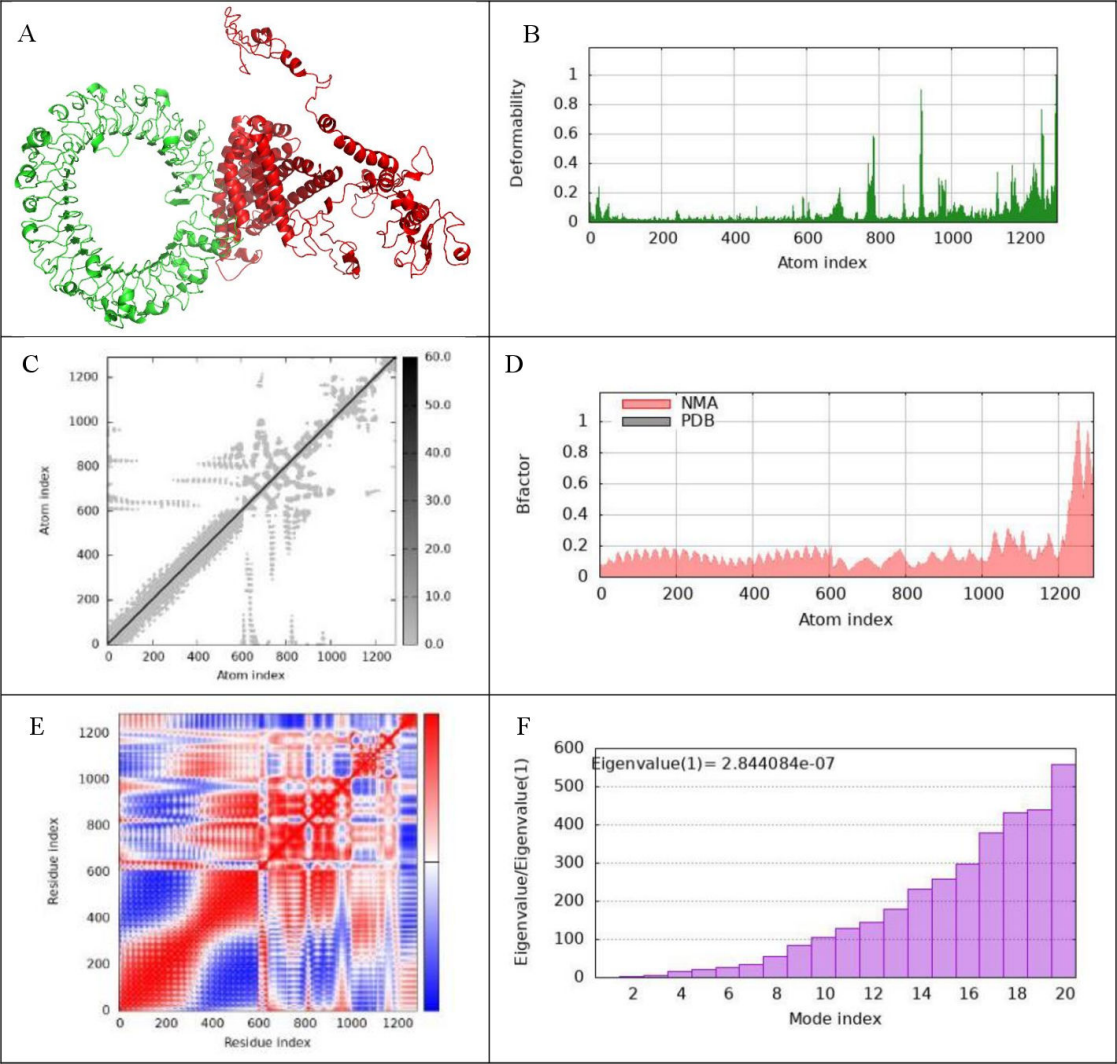
Examination of molecular dynamics simulations, normal mode analysis, and receptor-ligand interactions: (**A**) the docked configuration of the vaccine-TLR9 complex produced using the Cluspro server; (**B**) a graph depicting deformability; (**C**) an elastic network model generated via the iMODS server; (**D**) representation of B-factors; (**E**) covariance matrix; (**F**) eigenvalue related to the vaccine–TLR9 complex; (**G**) analysis of receptor–ligand interactions conducted through the PDBsum webserver; (**H**) visualization of receptor–ligand interactions performed using the LigPlot^+^. This figure offers a comprehensive analysis of molecular dynamics simulations, normal mode analysis, and receptor–ligand interactions associated with the vaccine–TLR9 complex, incorporating a variety of analytical components.

To investigate the interactions between the vaccine complexes and Toll-like receptors (TLRs) 2, 3, 4, and 9, we conducted comprehensive molecular dynamics simulations using the iMODS server. This analysis was complemented by an in-depth assessment of receptor–ligand interactions to elucidate the binding characteristics of the vaccine constructs. The deformability profiles of the constructs were illustrated through deformability graphs (Fig. 7B, 8B, 9B, and 10B), which highlighted specific amino acids exhibiting coiled conformations. These peaks signify regions of increased flexibility that may be crucial for effective receptor engagement. Additionally, normal mode analysis (NMA) was employed to characterize the intrinsic flexibility of the proteins. The B-factor graphs (Fig. 7D, 8D, 9D, and 10D) demonstrated a strong correlation between the NMA results and specific regions within the Protein Data Bank (PDB) structures of the uploaded complexes, revealing insights into their dynamic behavior. The eigenvalues for the docked complexes are presented in Fig. 7F, 8F, 9F, and 10F. Collectively, these analyses indicate that the vaccine–receptor complexes exhibit a low deformation index, considerable stiffness, and high stability, suggesting a robust structural integrity conducive to effective immune interactions. Fig. 7E, 8E, 9E, and 10E display the covariance matrix, which illustrates the relationships among pairs of amino acids distributed across dynamic regions of the protein. In this matrix, red highlights linked residues that exhibit correlated movements, while white indicates anti-correlated pairs, and blue denotes non-correlated residues. This information is critical for understanding how conformational changes in one part of the protein may affect other regions. Furthermore, a connectivity matrix based on the elastic network model was used to identify atom pairs linked by springs (Fig. 7C, 8C, 9C, and 10C). Each chain within the complex exhibited high stiffness, with darker gray areas indicating regions of increased rigidity. These findings suggest that the vaccine–receptor composite is characterized by strong intermolecular interactions and a firm structural framework. In conclusion, our results indicate that the vaccine–receptor complexes possess significant stability and rigidity, which may enhance their efficacy as a vaccine against *T. annulata*. The robust intermolecular interactions identified in this study underscore the potential of these constructs to effectively engage TLRs and stimulate an immune response.

## DISCUSSION

Veterinary vaccine development has typically followed a straightforward approach: isolating and inactivating pathogens to create candidates, resulting in successful modified live vaccines (MLVs). However, some diseases resist these conventional methods, prompting the exploration of alternatives like subunit vaccines, which require adjuvants and can be costly. Innovative technologies, such as recombinant vector and DNA vaccines, show promise but face efficacy and production challenges that hinder commercialization. Overall, while traditional methods have been effective, ongoing research aims to overcome their limitations and expand the strategies for developing effective veterinary vaccines (24, 59–61).

Numerous antigenic targets have been explored as probable components for constructing subunit vaccines against *Theileria* infections in ruminants. Notable examples include the sporozoite surface antigen SPAG-1, merozoite surface antigen Tams1, and *T. annulata* surface protein TaSp, all of which have shown the ability to confer partial protection against parasite challenges in both *in vitro* and *in vivo* investigational settings (14, 18).

In a study by Saaid et al. (14), the efficacy of recombinant *T. annulata* surface protein (TaSP) was evaluated for its potential to enhance the protective capacity of the attenuated Atbara cell line against field challenges in calves. Twenty-three crossbred calves were allocated into four groups, each receiving different vaccination protocols. After immunization and subsequent exposure to natural field tick infestations, all groups exhibited varying degrees of clinical theileriosis symptoms. Notably, the combination of the cell line and TaSP vaccination produced a synergistic effect, yielding a higher level of protection compared with individual vaccinations or the use of the cell line alone. This was evidenced by a 100% survival rate in the combined vaccine group (14).

DNA vaccines present a compelling strategy for the prevention of intracellular pathogens, such as *Theileria* parasites. These vaccines have shown a sturdy capacity to elicit cell-mediated immune responses, particularly by inducing T helper 1 cytokines, including IL-12, IFN-γ, TNF-α, and IL-21, which are essential for effective immune defense against various pathogens (1).

Fry et al. (62) were pioneers in demonstrating the particle-mediated epidermal delivery of a DNA vaccine. They introduced a *T. parva* polymorphic immunodominant molecule (PIM) antigen encoded in DNA, utilizing both codon-optimized and native sequences delivered via the intradermal route in Holstein steers. This antigen model was used to evaluate bovine GG immunization. Notably, immunization with the mammalian codon-optimized PIM sequence resulted in substantial anti-PIM antibody and cell-mediated immune responses in seven out of eight steers. However, no substantial differences were obtained between the immunized and control groups following a challenge with *T. parva*. These results suggest that the administration of PIM in this study did not confer adequate protection against *T. parva* in cattle. Nevertheless, the vigorous immune responses provoked against this model antigen indicate that GG immunization may serve as a talented vaccine for *T. parva* and other bovine pathogens (1, 62).

Attenuated, subunit, and DNA vaccines have limitations, but mRNA vaccines have shown efficacy against viral infections, like Zika, influenza, rabies, and coronaviruses. Increasingly recognized as safe and effective, mRNA vaccines induce transient antigen expression, triggering targeted immune responses. Their advantages include streamlined production, rapid development, and improved efficiency. The use of immunoinformatics in mRNA vaccine development represents a significant advancement in vaccination research, positioning these vaccines as a favored option for averting microbial infections and associated diseases (24, 63–67).

The development of vaccines aims to induce long-lasting immunity, necessitating the concurrent activation of both B and T cells. This dual activation is essential for an effective response to subsequent infections. Identifying epitopes that can stimulate both types of immune cells is critical for successful vaccine design. HTLs produce cytokines, such as IL-4, IFN-γ, and IL-10, which are pivotal in orchestrating immune responses. Antigen-presenting cells (APCs) play a central role by presenting epitopes to HTLs, which in turn secrete chemokines vital for combating pathogens. B cells, equipped with membrane-bound immunoglobulin receptors, recognize antigenic epitopes, leading to their dealing out and demonstration to T cells via MHC class II molecules. This interaction facilitates the differentiation of B cells into plasma cells, which secrete antibodies that neutralize both memory and foreign cells (24, 68, 69).

This study presents a significant advancement in the design of a novel mRNA vaccine targeting the surface proteins of *T.annulata*. The rigorous bioinformatics analysis conducted on the *Theileria* proteome was pivotal, as it successfully narrowed down 8,887 potential proteins to nine promising candidates. This selection process underscores the efficacy of our methodology and highlights the potential for these proteins to serve as effective targets for eliciting both humoral and cell-mediated immune responses. The use of the IEDB database for predicting LBL and CTL epitopes, coupled with B-cell epitope predictions via ABCpred, demonstrates our commitment to a comprehensive approach. The assessment of these epitopes for antigenicity, allergenicity, and toxicity through web-based servers further validates their suitability for vaccine development. The inclusion of specific linkers to connect these epitopes enhances the structural integrity and functionality of the vaccine construct. Our *in silico* immune simulation results are particularly promising, revealing that the CTL epitopes correspond to seven MHC alleles in cattle. This finding suggests that the vaccine is well-positioned to effectively stimulate an immune response in the target population. The molecular docking analyses performed using the ClusPro platform showed strong binding affinities between the selected epitopes and their respective MHC alleles, reinforcing the potential effectiveness of our proposed vaccine. Moreover, the design of the mRNA construct, incorporating elements, such as the 5′ m7G cap sequence, globin 5′ and 3′ UTRs, poly(A) tails, a Kozak sequence, and a stop codon, reflects a strategic approach to enhance both stability and translation efficiency. This meticulous design is essential for ensuring that the vaccine can elicit robust immune responses. The evaluation of binding affinities with immune receptors TLR-2, TLR-3, TLR-4, and TLR-9 further supports our findings, indicating that the vaccine construct is poised to effectively stimulate both innate and adaptive immune responses. Such dual activation is crucial for achieving long-lasting immunity against *T. annulata* infection. Additionally, molecular dynamics simulations confirmed the stability of the mRNA vaccine complex, while immunoinformatics approaches established its characteristics as steady, thermostable, antigenic, non-allergenic, and hydrophilic. This stability is critical for practical applications in vaccine delivery and storage. We anticipate that the proposed vaccine will elicit an immunological response following three injections, promoting the generation of memory cells essential for long-term protection. The observed activity of macrophages and dendritic cells, along with calculations using the Simpson index, indicating memory cell presence, further corroborates our expectations for an effective immune response.

One of the limitations encountered in this study was restricted access to the structures of MHC alleles, as well as a scarcity of servers primarily designed for human analysis. Additionally, we recommend that future validation efforts be conducted in laboratory settings or through animal model studies.

In conclusion, this comprehensive study not only lays a solid foundation for an innovative mRNA vaccine against *T. annulata* but also highlights its considerable promise in protecting this significant pathogen affecting cattle. The results underscore the potential impact of this research on veterinary medicine and livestock health management.

### Conclusions

The results of this investigation suggest that the proposed multi-epitope mRNA vaccine could serve as an effective strategy for addressing infections caused by *T. annulata*, but further *in vitro* and *in vivo* research is required to authorize its safety and efficacy.
