# Erratum for Fattahi and Sadeghi Kalani, “mRNA vaccine design using the proteome of *Theileria annulata* through immunoinformatics approaches”

**DOI:** 10.1128/msphere.00665-25

**Published:** 2025-09-30

**Authors:** Roohollah Fattahi, Behrooz Sadeghi Kalani

## ERRATUM

Volume 10, no. 5, e00809-24, 2025, https://doi.org/10.1128/msphere.00809-24. Page 1: The corresponding author’s email address should read “r.fattahi@ilam.ac.ir.”

